# A Trailblazer in Anatomy: Madhusudan Gupta and the Historic First Dissection

**DOI:** 10.7759/cureus.70612

**Published:** 2024-10-01

**Authors:** Deepa G, Mrudula Chandrupatla, Shrikrishna B H

**Affiliations:** 1 Anatomy, All India Institute of Medical Sciences, Bibinagar, Hyderabad, IND; 2 Otorhinolaryngology Head-Neck Surgery, All India Institute of Medical Sciences, Bibinagar, Hyderabad, IND

**Keywords:** cadaver dissection, health professionals education, india, medical edu, professional ethics, social and cultural anthropology

## Abstract

The life and accomplishments of Madhusudan Gupta, a significant person in Indian medical history, are discussed in this review article. Born into an aristocratic Bengali family, Gupta initially showed little interest in formal education. However, his enrolment in Sanskrit College and subsequent involvement with Calcutta Medical College (CMC) marked a turning point in his life. Under European guidance, Gupta challenged deep-rooted societal and religious taboos by conducting the first cadaveric dissection in India, a groundbreaking act that significantly advanced anatomical studies in the country. Facing severe opposition, Gupta utilized his profound knowledge of Sanskrit texts to justify the practice of dissection, helping integrate Western medical practices into Indian education. His work not only revolutionized medical education in India but also paved the way for future reforms in the field. Gupta's legacy is honoured through various accolades, including the Pandit Madhusudan Gupta Memorial Lifetime Achievement Award, underscoring his crucial role in the development of modern anatomy and medicine in India.

## Introduction and background

The history of anatomy is deeply intertwined with the development of medicine and society over the centuries. From ancient times to modern advancements, the study of anatomy has been fundamental to medical education and practice. The evolution of anatomical knowledge has not only shaped medical understanding but has also raised ethical considerations and influenced societal norms.

The history of anatomy is not just a chronological progression of knowledge but also a reflection of ethical dilemmas and societal influences. Integrating insights from the history of anatomy into medical education can provide a holistic approach that emphasizes the shared humanity between medical practitioners and patients [1]. By understanding the historical context of anatomy, educators and clinicians can appreciate the ethical responsibilities associated with studying the human body and disseminating anatomical knowledge [2].

Henry Gray, known for his work "Gray's Anatomy," also played a crucial role in advancing anatomical knowledge. His comprehensive anatomical textbook has been a cornerstone in medical education, providing detailed insights into the structure and function of the human body [3]. Andreas Vesalius, known as the Father of Modern Anatomy, revolutionized the study of anatomy by emphasizing the importance of dissection and experimental observation [4]. His meticulous anatomical descriptions and precise illustrations in his work "De Humani Corporis Fabrica" laid the foundation for modern anatomical studies [5]. Similarly, Sushruta, regarded as the Father of Indian Surgical History, made remarkable contributions to the field of anatomy through his work in ancient Indian texts such as the "Sushruta Samhita" [6].

The legacy of Madhusudan Gupta stands as a towering figure in the annals of Indian medical history. He was an Ayurvedic physician. His courage in performing India's first human dissection in 1836, a time fraught with religious and social taboos, was a watershed moment [7]. This audacious act laid the foundation for modern medical education in India. Gupta's legacy extends far beyond this groundbreaking achievement; his work as a translator, educator, and medical reformer has left an enduring impact on Indian healthcare [8].

This review delves into the life and work of this extraordinary individual, exploring his pivotal role in shaping the trajectory of anatomy and medicine in India. His work has not only advanced the field of anatomy during their respective times but continues to influence modern medical education and practice.

## Review

Early journey of life

Madhusudan Gupta (1800-1856) came from an aristocratic background. Madhusudan Gupta was descended from a Vaidya family in the Hooghly district village of Baidyabati, India. High in the feudal hierarchy were his ancestors. His grandfather served as the "family physician to the Nawabs of Hooghly, who were local rulers in the Bengal region during the Mughal period in India," while his great-grandfather bore the honorific "Bakshi." The honorific "Bakshi" was a title of respect traditionally conferred upon individuals who were responsible for overseeing financial or military affairs in feudal settings. Unlike his ancestors, Madhusudan showed no early interest in a formal education. It is stated that his father became enraged due to his dislike of studying and threatened to throw him out of the house. His father’s attitude made Madhusudan to move out [9].

In December 1826, he was enrolled in the Sanskrit College's recently established ayurvedic course. Madhusudan rose to the top of the class and demonstrated remarkable aptitude in this curriculum. He was promoted from the position of student to the chair of teaching in May 1830. He stayed in that position until January 1835 [9].

The Calcutta Medical College (CMC) milestone: a path to medicine

By proclamation of Lord William Bentinck who was the first Governor General of India, a new era began on January 28, 1835. The "new era" refers to the introduction of Western medical education in India, starting with the founding of CMC. This marked a significant shift toward formalized, European-style medical instruction, including dissection, challenging societal taboos, and modernizing India's approach to medicine and healthcare. With the appointment of Assistant Surgeon M.J. Bramley as the first Principal of Medical College Hospital (MCH), the first medical college in Asia was established [10].

On March 17, 1835, Madhusudan Gupta joined CMC as a native teacher [9]. For a four-year period, 50 young men between the ages of 14 and 20 were permitted to study without regard to their caste or creed. The language of instruction was English, and rigorous adherence to European medical education protocols was maintained. Thus, the door to Western medical education opened for the first time in the East [10].

Henry Goodeve served as an assistant surgeon for the East India Company's Bengal Principality in 1831 and as assistant to Dr. MJ Bramley, in 1835, who was the Superintendent of the CMC [11]. With 350 patient beds, the MCH Building was formally inaugurated in 1852. It is the most recognizable image of this illustrious institution and the oldest building on the premises. Dr. Henry Goodeve was the first professor of medicine. The University of Calcutta was established in 1857, and Medical College was affiliated to it [10].

The first dissection: Madhusudan Gupta's pioneering role in modern anatomy

As the traditional Indian Hindu prejudice against human dissection was a significant issue, the CMC sought assistance from Dwarkanath Tagore and Ram Mohan Roy. These and other Indians agreed to support dissection only if it could be found in the Indian traditional literature. Later, Madhusudan Gupta was given the task of locating such authority in the Sanskrit texts [11].

**Figure 1:**
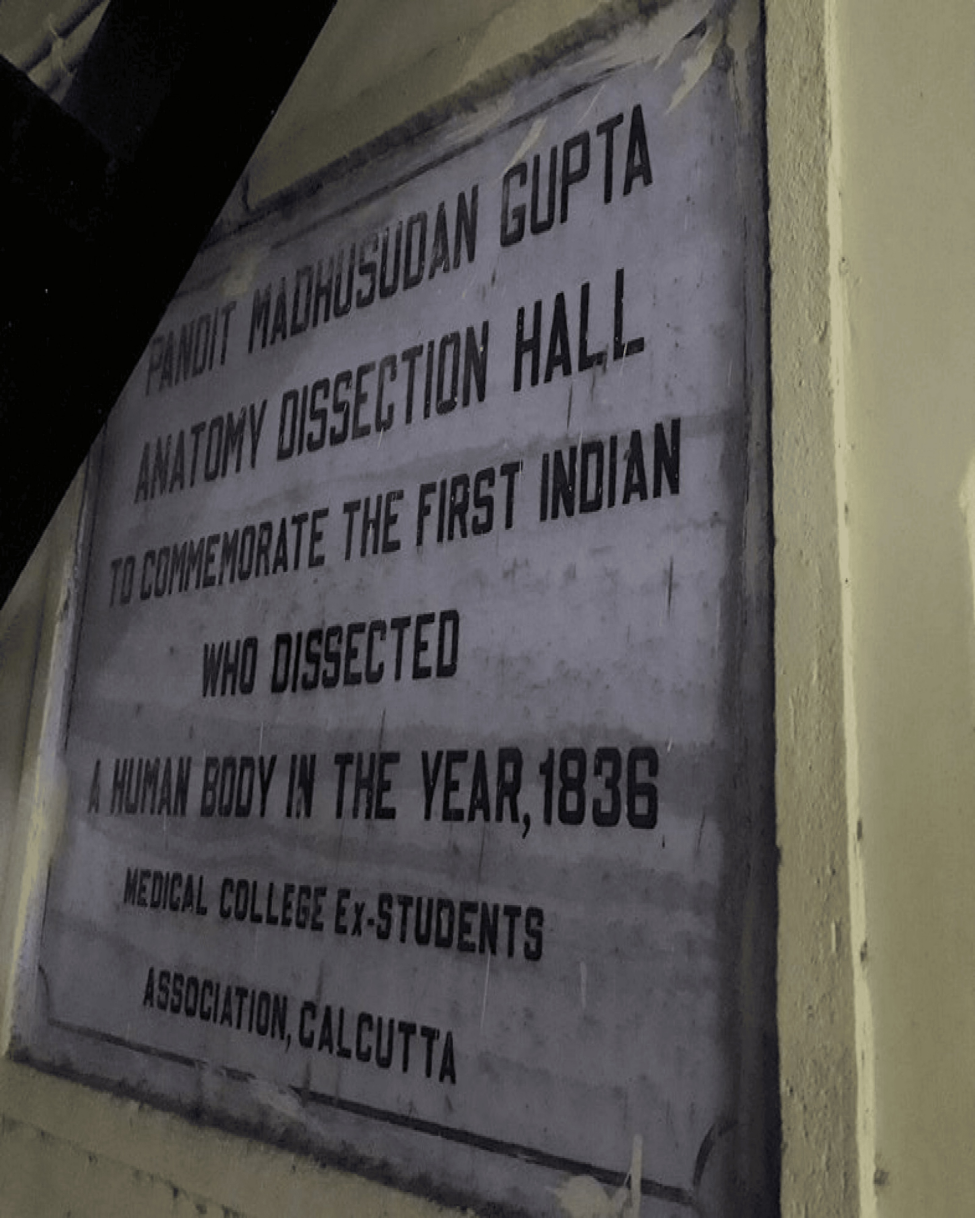
Plaques in the memory of the first dissection of a human corpse at Calcutta Medical College. This image was sourced from Wikimedia Commons and is attributed to Pinakpani. The image is licensed under the Creative Commons Attribution-ShareAlike 4.0 International License. Source: Pinakpani (https://commons.wikimedia.org/wiki/File:Plaques_in_the_memory_of_first_dissection_of_a_human_corpse_in_CMC_01.jpg), https://creativecommons.org/licenses/by-sa/4.0/legalcode

One of the biggest obstacles to overcome was the pervasive prejudice against studying anatomy and dissection, but this was also surmounted on January 10, 1836, when Madhusudan Gupta, performed the first cadaveric dissection under Dr. Goodeve's supervision [9]. It was the first human dissection in Asia. Reports state that he received help from four brave students - Umacharan Set, Rajkrishna De, Dwarakanath Gupta, and Nabin Chandra Mitra [12]. The year 1836 marked a significant milestone in Asian medical history, as Madhusudan Gupta conducted the first human dissection on the continent. Fort William, the British outpost in Kolkata, is reported to have honoured the accomplishment with a 50-gun salute. Seeing a person from the highest caste dissect a dead body was a momentous occasion [13]. Following this widely reported occurrence in Britain as a triumph of Western "superiority," a portrait of Gupta was shown in the CMC a few years later, and it remains there to this day [14]. CMC marks the site of the first human dissection (Figure 1).

Following the groundbreaking human dissection of 1836 at the Medical College and Hospital in Kolkata, the conservative Hindu society in Calcutta vehemently opposed this act, deeming it sacrilegious. Madhusudan Gupta, a Brahmin involved in the dissection, was subsequently ostracized for his actions. However, Gupta skillfully countered these protests by citing ancient Sanskrit texts that supported the practice of human dissection in ancient India. His victory over the conservative leaders, facilitated by his deep knowledge of the scriptures, has been attributed to his scholarly prowess. According to Sundarimohan Das, the then-prominent Indian physician and social reformer, known for his contributions to medical education and public health, the debate was organized at the initiative of J.E.D. Bethune, the Lieutenant Governor of Bengal, with the Maharaja of Navadwip presiding over the conference [15].

Madhusudan Gupta's pioneering efforts paved the way for the acceptance of human dissection in India, overcoming significant societal and religious obstacles. The number of human bodies dissected at the Medical College and Hospital in Kolkata surged dramatically between 1837 and 1844, increasing from 60 in 1837 to over 500 in 1844. In total, nearly 3,500 bodies were dissected during this eight-year period [11].

The final years: lasting influence and legacy

**Figure 2:**
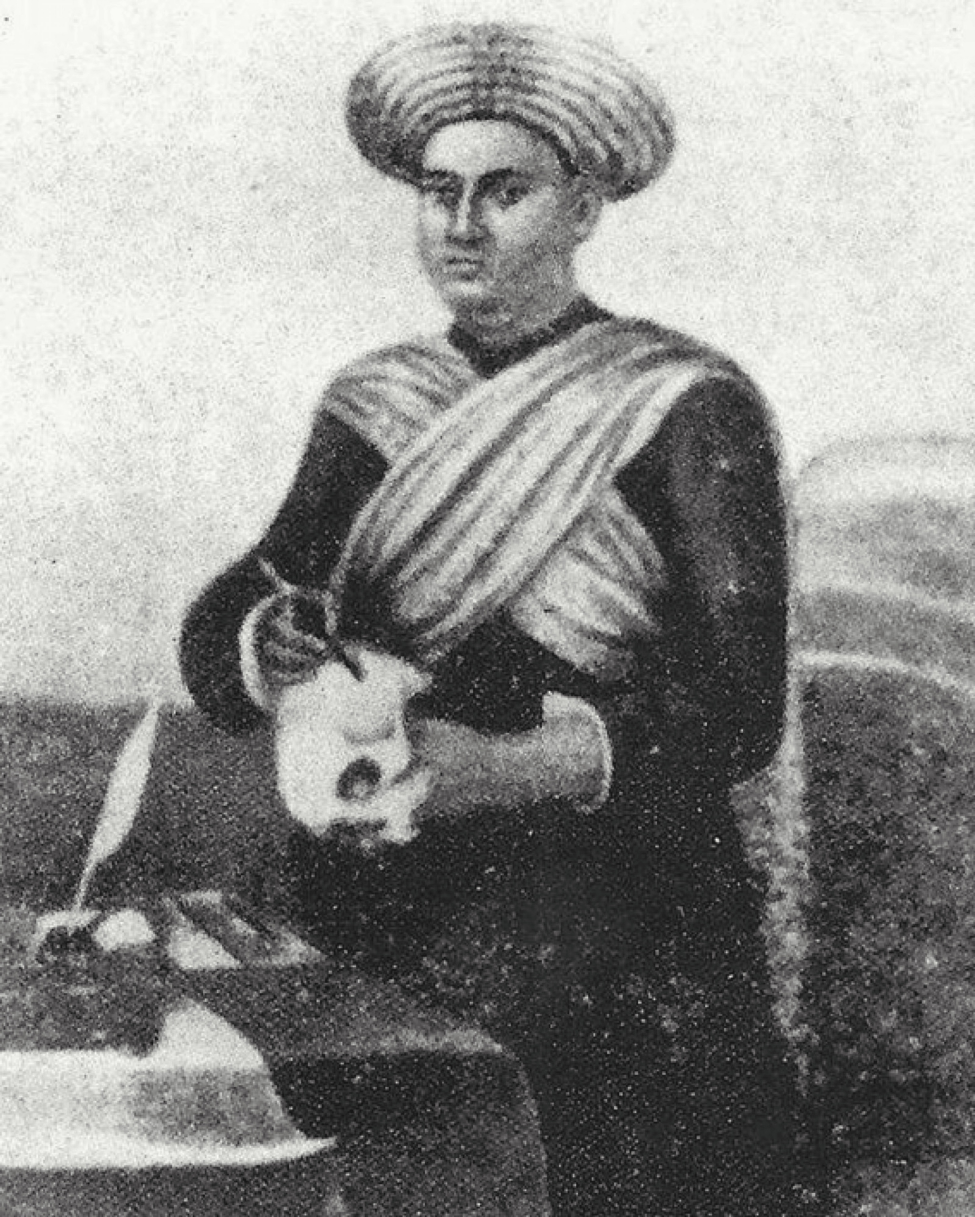
Madhusudan Gupta. Scanned image of a photo reprint of an oil painting by S.C. Belnos, the wife of Jean Jacques Belnos, presented to Medical College by Drinkwater Bethune in 1850. The artwork is associated with the Bengal Renaissance and is an important piece reflecting the cultural and historical significance of that era. Source: Wikimedia Commons, 1850. The image is in the public domain under Indian law (published before 1964) and likely in other jurisdictions with similar copyright terms. No permission is required for reproduction, and it can be freely used in academic publications.

Madhusudan Gupta, a pioneer in medical education in India, died due to diabetic septicemia on November 15, 1856. In recognition of his contributions, J.E.D. Bethune presented a portrait of Gupta, painted by Mrs. Belnos, to the MCH (Figure 2) in Kolkata in 1850 [12].

In recognition of his significant contributions to the field of anatomy, the prestigious Pandit Madhusudan Gupta Memorial Lifetime Achievement Award was established in recent years. As a testament to his own exceptional achievements, Dr. A. K. Datta was honoured with this award in 2008 by the West Bengal Chapter of the Anatomical Society of India [16].

## Conclusions

In conclusion, the history of anatomy is not just a narrative of scientific progress but a reflection of the intertwined relationship between medicine, ethics, and society. Madhusudan Gupta's legacy is a testament to his unwavering pursuit of knowledge and his dedication to the advancement of medical science in India. His pioneering efforts in human dissection and his contributions to medical education and reform have left an indelible mark on the nation's healthcare landscape.
